# Novel magnetic resonance imaging marker of diffuse myocardial fibrosis in hypertensive heart disease: the role of transcytolemmal water-exchange

**DOI:** 10.1186/1532-429X-14-S1-O115

**Published:** 2012-02-01

**Authors:** Otavio R Coelho-Filho, Richard N Mitchell, Heitor Moreno, Raymond Kwong, Michael Jerosch-Herold

**Affiliations:** 1 Medicine, State University of Campinas, Campinas, Brazil; 2 Medicine, Brigham and Women's Hospital, Boston, MA, USA; 3 Radiology, Brigham and Women's Hospital, Boston, MA, USA

## Summary

Transcytolemal water exchange and its effect on myocardial T1 relaxation can, if neglected, lead to a significant underestimate of the myocardial extracellular volume fraction, a novel marker of diffuse fibrosis.

## Background

LGE may fail to detect diffuse fibrosis in several cardiac conditions. A novel approach uses the myocardial extracellular volume-fraction (MECVF), measured as distribution volume of a gadolinium constrast, as a marker of extracellular expansion. Previous studies have assumed the fast-exchange (FX) limit for the transcytolemal water-exchange, yet the administration of an extracellular-agent can create significant transcytolemal T1-differences, and cause an underestimation of extracellular volume fraction under the FX assumption. We hypothesized that the quantitative measure of MECVF with using a 2-site H-exchange model (2SX-model) correlates positively with the extracellular volume fraction, while the FX approach underestimates extra-cellular matrix expansion in a rodent model of hypertensive heart disease and diffuse myocardial fibrosis created by administration of L-NAME.

## Methods

L-NAME(3mg/ml) or placebo was administered respectively to 22(bw=36.9±2.3g) and 15(bw=37.6±2.5g) wild-type mice. Animals were imaged at baseline and 7-weeks after treatment on a 4.7T small-animal MRI-system. T1(#of T1’s>5/mouse) was quantified with a modified Look-Locker gradient-echo-cine technique, before and after fractionated Gd-DPTA administration(mean max. R1 in blood post-contrast=5.0±2.26 1/s). MECVF obtained from the T1 measurements with the 2SX and FX-models, and by using blood hematocrit to adjust the partition coefficient. Connective tissue volume fraction (CTVF) was measured using Masson’s trichrome.

## Results

**Figure 1:**
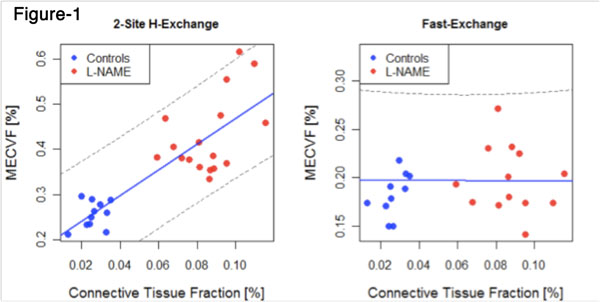
Correlation: MECVF (2-site H-exchange and Fast-exchange) and connective tissue volume fraction

**Figure 2:**
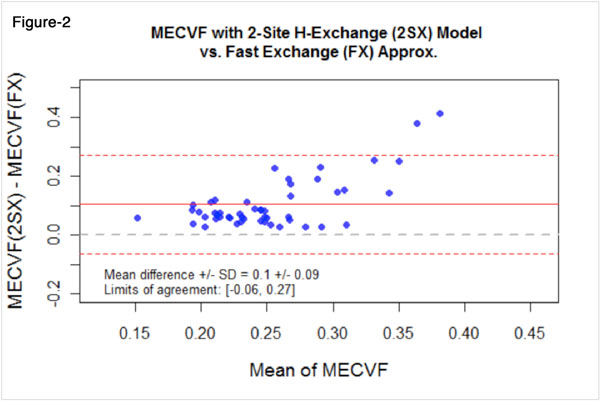
Bland-Altman analysis

L-NAME-treated animals demonstrated hypertrophy (weight-indexed LVmass 4.1±0.4 vs. 2.2±0.3 μg/g, p<0.001) and increased CTVF (8.6%±1.5 vs. 2.58%±0.6, P<0.001) as compared to controls. MECVF was substantially higher in L-NAME-treated animals (0.43±0.09 vs, 0.26±0.03, p<0.001 with 2SX-model; 0.20±0.03 vs. 0.20±0.05, p=0.82 without TWE with FX assumption). MECVF from 2SX-model showed a good correlation with CTVF and weight-indexed LVmass (both r=0.8, p<0.0001), while MECVF from FX-model did not correlate significantly with CTVF(p=0.44) and LVmass(p=0.80)(fig-1). MECVF from the 2SX model also correlated with the LVEF at 7-weeks(r=0.48, p=0.01). Bland-Altman analysis demonstrated that neglect of the transcytolemal water exchange causes a significant underestimate MECVF expansion(fig-2), which worsens with extracellular matrix expansion and LVH.

## Conclusions

CMR MECVF quantification provides a robust measure of myocardial extracellular matrix expansion and interstitial fibrosis, though any break-down of the FX assumption for transcytolemmal exchange can result in a significant underestimation of MECVF. Importantly, underestimates of MECVF due to the FX assumption depend on the degree of cell-hypertrophy, and the maximum T1 in the blood pool. A break-down of the FX assumption cannot be detected with protocols limited to a pair of pre/post-contrast T1 measurements. A generalization of the model for determination of MECVF brings important benefits for an early detection of diffuse fibrosis.

## Funding

Supported by the American Heart Association (AHA 11POST5550053) and the National Institutes of Health/NHLBI (1R01HL090634-01A1).

